# Aptamer’s Structure Optimization for Better Diagnosis and Treatment of Glial Tumors

**DOI:** 10.3390/cancers16234111

**Published:** 2024-12-08

**Authors:** Anastasia A. Koshmanova, Polina V. Artyushenko, Irina A. Shchugoreva, Victoriya D. Fedotovskaya, Natalia A. Luzan, Olga S. Kolovskaya, Galina S. Zamay, Kirill A. Lukyanenko, Dmitriy V. Veprintsev, Elena D. Khilazheva, Tatiana N. Zamay, Daria A. Ivanova, Maria R. Kastyuk, Ivan N. Lapin, Valery A. Svetlichnyi, Felix N. Tomilin, Nikita A. Shved, Valeriia S. Gulaia, Vadim V. Kumeiko, Maxim V. Berezovski, Anna S. Kichkailo

**Affiliations:** 1 Laboratory for Digital Controlled Drugs and Theranostics, Federal Research Center “Krasnoyarsk Science Center SB RAS”, 660036 Krasnoyarsk, Russia; koshmanova.1998@mail.ru (A.A.K.); art_polly@mail.ru (P.V.A.); shchugorevai@mail.ru (I.A.S.); viktoriia.fedotovskaia@yandex.ru (V.D.F.); zamaykin@mail.ru (O.S.K.); gzamay@yandex.ru (G.S.Z.); k.a.lukyanenko@yandex.ru (K.A.L.); d_veprintsev@mail.ru (D.V.V.); tzamay@yandex.ru (T.N.Z.); dariaivanova9521@yandex.ru (D.A.I.); mashakastyuk@yandex.ru (M.R.K.); felix@iph.krasn.ru (F.N.T.); 2 Laboratory for Biomolecular and Medical Technologies, Krasnoyarsk State Medical University, 660022 Krasnoyarsk, Russia; laskimo@mail.ru; 3 Research Institute of Molecular Medicine and Pathobiochemistry, Krasnoyarsk State Medical University, 660022 Krasnoyarsk, Russia; elena.hilazheva@mail.ru; 4 Laboratory of Physics of Magnetic Phenomena, Kirensky Institute of Physics, 660036 Krasnoyarsk, Russia; 5 Laboratory of Advanced Materials and Technology, Siberian Physical-Technical Institute of Tomsk State University, 634050 Tomsk, Russia; 201kiop@mail.ru (I.N.L.);; 6 School of Medicine and Life Sciences, Far Eastern Federal University, 690922 Vladivostok, Russia; nikitawayfarer@yandex.ru (N.A.S.); gulaia.vs@dvfu.ru (V.S.G.); vkumeiko@yandex.ru (V.V.K.); 7 A.V. Zhirmunsky National Scientific Center of Marine Biology, Far Eastern Branch of Russian Academy of Sciences, 690041 Vladivostok, Russia; 8 Department of Chemistry and Biomolecular Sciences, University of Ottawa, Ottawa, ON K1N6N5, Canada

**Keywords:** glial brain tumors, aptamer binding, molecular modeling, molecular structure, aptamer internalization

## Abstract

**Background:** Oncological diseases are a major focus in medicine, with millions diagnosed each year, leading researchers to seek new diagnostic and treatment methods. One promising avenue is the development of targeted therapies and rapid diagnostic tests using recognition molecules. The pharmaceutical industry is increasingly exploring nucleic acid-based therapeutics. However, producing long oligonucleotides, especially aptamers, poses significant production challenges. **Objectives:** This study aims to demonstrate the efficacy of using molecular modeling, supported by experimental procedures, for altering aptamer nucleotide sequences while maintaining their binding capabilities. The focus is on reducing production costs and enhancing binding dynamics by removing nonfunctional regions and minimizing nonspecific binding. **Methods:** A molecular modeling approach was employed to elucidate the structure of a DNA aptamer, Gli-55, facilitating the truncation of nonessential regions in the Gli-55 aptamer, which selectively binds to glioblastoma (GBM). This process aimed to produce a truncated aptamer, Gli-35, capable of forming similar structural elements to the original sequence with reduced nonspecific binding. The efficiency of the truncation was proved by flow cytometry, fluorescence polarization (FP), and confocal microscopy. **Results:** The molecular design indicated that the new truncated Gli-35 aptamer retained the structural integrity of Gli-55. In vitro studies showed that Gli-35 had a binding affinity comparable to the initial long aptamer while the selectivity increased. Gli-35 internalized inside the cell faster than Gli-55 and crossed the blood–brain barrier (BBB), as demonstrated in an in vitro model. **Conclusions:** The success of this truncation approach suggests its potential applicability in scenarios where molecular target information is limited. The study highlights a strategic and resource-efficient methodology for aptamer development. By employing molecular modeling and truncation, researchers can reduce production costs and avoid trial and error in sequence selection. This approach is promising for enhancing the efficiency of therapeutic agent development, particularly in cases lacking detailed molecular target insights.

## Simple Summary

This research focuses on finding more efficient and cost-effective methods for the development of therapeutic agents to treat malignant tumors. The study explores the design of special molecules called aptamers, which can bind to cancer cells and help in diagnosis and treatment. However, creating these molecules can be expensive and technically challenging. Using computer modeling, a way was found to simplify these molecules, reducing unnecessary parts while keeping their effectiveness intact. This method not only cuts down on production costs but also improves how quickly and precisely these molecules can work within the body. This approach promises to benefit researchers in developing targeted cancer therapies, even when detailed information about the cancer targets is lacking.

## 1. Introduction

The fight against malignant tumors has become one of the central themes in medicine. Millions of people are diagnosed with this frightening disease every year, prompting researchers to seek new methods for its diagnosis and treatment. One promising direction is the development of targeted therapies and precise, rapid test systems for tumor cells or tumor biomarker molecules. Single-stranded DNA or RNA aptamers, which are analogs of monoclonal antibodies, are highly effective in identifying targets both within the body and in analyzed biofluids. Aptamers can be utilized for the delivery of various drugs, making them excellent delivery vehicles.

Single-stranded DNAs and RNAs obtained through the SELEX (systematic evolution of ligands by exponential enrichment) process consist of 80 to 100 nucleotides (nt), including the primer sites. It is important to note that only a portion of nucleotides in each aptamer sequence are responsible for the binding functionality of the aptamers. Therefore, each aptamer sequence is composed of two key regions: an essential region and a nonessential region [1,2]. The essential nucleotides that interact with the targets typically range from 25 to 40 nt in length and exhibit structures such as hairpin loops, G-quartets, bulges, or pseudoknots [3]. Often, nonessential nucleotides can diminish the binding affinity of the full-length aptamer. Thus, removing these regions may either have no effect or may, in some cases, significantly enhance binding affinity. The most prevalent methodology for the truncation of aptamers subsequent to the completion of the selection process is a trial-and-error approach, which is frequently both time-consuming and laborious [4]. Therefore, there is an increasing tendency to truncate aptamers based on their secondary structure [5,6]. Currently, there is no standardized algorithm for aptamer truncation based on spatial conformation.

For the design of any diagnostic reagents or therapeutic drugs, it is essential to obtain aptamers with minimal nonspecific binding and a well-established mechanism of action. In our previous study, we selected aptamers for glial brain tumors using conventional cell-SELEX [7]. Gli-55 aptamer demonstrated good binding to tumor cells but was found to bind to glial fibrillary acidic protein (GFAP). Thus, it has a good potential to be applied as a visualization probe or for drug delivery.

The core technology to obtain an aptamer-based drug is its synthesis, which highly depends on the length of the oligonucleotide. Solid-phase synthesis technology presents significant challenges in terms of expanding production scale while maintaining decent quality. As market demand grows, achieving high yield along with precise synthesis accuracy, reduced production time, and enhanced purity has become a crucial challenge in the development and commercialization of aptamer-based medicines.

Truncation of an aptamer longer than 50 nt may facilitate synthesis and improve product quality and yield. This approach can also enhance the binding speed and increase its permeability across cell membranes and the BBB. Here, we present an algorithm for improving aptamer conformation through hairpin structural optimization.

## 2. Materials and Methods

### 2.1. Molecular Modeling

Prediction of the secondary structure for the aptamers was performed using the mFold web server [8]. The free energy of the aptamer folding and the details of the energetic contributions for each loop were also obtained with this program. The 3D model of the aptamers was designed using the SimRNA and VMD programs [9,10,11]. Molecular dynamic simulations were performed using the GROMACS package [12]. Simulations were performed using periodic boundary conditions, Amber14sb force field [13], and a TIP3P model for water [14]. The negative charge of the aptamer was neutralized by adding sodium ions. Extra sodium and chloride ions were added to a rich 0.15 mM concentration to imitate the conditions of in vitro experiments. In total, 200 ns molecular dynamics (MD) simulations were performed, and clustering analysis of the obtained MD trajectories was performed using the quality threshold algorithm implemented in VMD [15].

### 2.2. Tumor Samples

The research received ethical approval from the Local Ethics Committee of Krasnoyarsk State Medical University in Krasnoyarsk, Russia (reference number 37/2012 dated 31 January 2012), as well as from the Local Ethics Committee of the Krasnoyarsk Inter-District Ambulance Hospital, named after N.S. Karpovich (20 November 2016). Tumor samples were obtained from a patient diagnosed with glioma who had undergone complete curative surgical removal of the tumor at the Krasnoyarsk Inter-District Ambulance Hospital. The collection of brain tumor specimens was conducted with the patient’s written informed consent. The tissues were excised under aseptic conditions and were promptly placed into ice-cold, colorless Dulbecco’s modified Eagle’s medium (DMEM) enriched with penicillin G at a concentration of 1000 U/mL and streptomycin at 1000 mg/L. The samples were then transported on ice to the laboratory within 2 to 4 h post surgery.

### 2.3. Primary Glial Tumor Cell Cultures and GBM Cell Lines

Tumor specimens underwent rinsing in antibiotic-supplemented DMEM, followed by the mincing of the tissues while removing blood vessels, necrotic material, and clots. The tissues were dissociated into small aggregates through pipetting and washed three times with Dulbecco’s phosphate-buffered saline (DPBS) via centrifugation at 3000 rpm. The resulting pellet was then transferred to plates with initiation medium (DMEM, 10% fetal bovine serum, 20 μg/mL insulin, 10 μg/mL transferrin, 25 nmol/L sodium selenite, 100 U/mL antibiotics, 1 ng/mL epidermal growth factor) and incubated in a humidified environment at 37 °C with 5% CO_2_. The cells were cultured for a month to achieve a confluent monolayer, with medium changes three times a week.

The human GBM U87MG cell line (ATCC, USA) and its genetically modified derivatives were used for the investigation of aptamer interactions with the most important glioma cell genotypes. Using clustered regularly interspaced short palindromic repeats (CRISPR)-Cas9 protocol with cytidine base editors, the most important polymorphisms in the IDH1 and TP53 genes typically occurring in glioma subtypes were introduced to U87MG glioma cells initially characterized as wild-type (WT) genetic variants. The base editor plasmids pBT277 and pBT375 were employed to induce TP53 R248Q polymorphism, while pEF-BFP and pEF-AncBE4max were utilized to induce the IDH1 R132H variant. U87MG-IDH1htz, which harbors the heterozygous (htz) R132H mutation in the IDH1 gene; U87MG-TP53hmz, carrying the homozygous (hmz) R248Q mutation in the TP53 gene; and U87MG-IDH1htz-TP53hmz, which contains both the heterozygous R132H mutation in the IDH1 gene and the homozygous R248Q mutation in the TP53 gene were utilized for aptamer binding experiments. All cell lines were cultured in DMEM media supplemented with L-Glutamine (Capricorn Scientific, GmbH, Ebsdorfergrund, Germany), along with 10% fetal bovine serum (FBS), 100 U/mL penicillin, and 100 μg/mL streptomycin. The cell lines were maintained at 37 °C in a humidified environment containing 5% CO_2_.

### 2.4. Primary Culture of Cerebral Endothelial Cells, Astrocytes, and Neurons

This study was conducted in strict accordance with the National Institute of Health Guidelines recommendations for the care and use of laboratory animals. The protocol was approved by the Local Committee on the Ethics of Experiments on Animals of the Krasnoyarsk State Medical University (number #115/2022 from 28 November 2022). All operations were performed under anesthesia, and every effort was made to minimize the animals’ suffering.

Progenitor cells for obtaining neurons and astrocytes were collected from the brains of newborn rats and cultured to form neurospheres. Neurospheres were differentiated into neurons and astrocytes. Endothelial cells were isolated from the vessels of the rat brain parenchyma (P10).

All procedures were performed on ice under sterile conditions. The brain of 7-day-old Wistar rats, from which progenitor cells were isolated using a previously developed protocol, followed by directed differentiation of progenitor cells into astrocytes and neurons to form a cellular ensemble [16]. The isolation and obtaining of the primary culture of cerebral endothelial cells from rat brain microvessels were carried out in accordance with the protocol for isolating and culturing cerebral endothelial cells [17].

### 2.5. Aptamer Synthesis

Fluorescein amidite (FAM)- and Cyanine5 (Cy5)-labeled aptamers Gli-100 5′-CTC CTC TGA CTG TAA CCA CGG TCC GGT TCA CCT CTA GCA TTC CTG GCG TTA TTA ACG GAG CAG TCC TGT GGA GTG GGT GACG CAT AGG TAG TCC AGA AGC -3′and Gli55 5′-GTCCG GTTCA CCTCT AGCAT TCCTG GCGTT ATTAA CGGAG CAGTC CTGTG GAGTG GGTGA-3′were purchased from Integrated DNA Technologies (IDT, Coralville, Iowa).

Other aptamers Gli35 5′-GCGTT ATTAA CGGAG CAGTC CTGTG GAGTG GGTGA-3′ and scrambled ScrGli-35 5′-TCGTC ATCGC GTGCG TCGGC GAATT AGTAG ATTTG-3′ were synthesized in the laboratory using an ASM-2000 high-throughput DNA/RNA synthesizer by BiosSet (BiosSet, Novosibirsk, Russia). An argon atmosphere was maintained within the synthesizer chamber, and the synthesis was carried out through the stepwise delivery of phosphoramidites dissolved in acetonitrile onto a carrier in a 96-well plate. Moisture from the chamber and acetonitrile was removed using zeolites (pore size 4 Å). The minimum concentration of water in the acetonitrile reached up to 30 ppm, as determined by a Karl Fischer titrator.

Following the synthesis, AMA (30% ammonia and 40% methylamine 1:1) was added and allowed to thermoregulate to facilitate deblocking; the resulting samples were filtered, dried, and cooled. Reprecipitation was then performed, followed by redissolving in water. Optical properties were checked using a Nano-500 spectrophotometer (Allsheng, Hangzhou, China) to control the yield. The samples were cooled for approximately 2 h and dried in a FreeZone lyophilizer (Labconco, Kansas, MO, USA).

To attach fluorescent labels Cy5 and FAM to the 5′ prime of the aptamers, click chemistry was employed. The dry labels were dissolved in dimethyl sulfoxide (DMSO). The sample was cooled, then placed in a lyophilizer at 45 °C for 4 h. The oligonucleotides were dissolved in water, a copper catalytic buffer was added and mixed, and then the calculated volume of azide dye solution was added. The mixture was thoroughly mixed again, degassed to remove oxygen, and a freshly prepared solution of the activator—ascorbic acid—was added. The test tube was purged with inert gas for a few minutes, sealed, and mixed again on a shaker. The mixture was held at room temperature for 10 h.

Post reaction, the resultant oligonucleotides were purified using an ACTA Pure chromatographic system (Cytiva). The sample was dried, reprecipitated, and analyzed using a Nano-500 spectrophotometer (Allsheng) and an Agilent Infinity 1260 liquid chromatograph with a qTOF 6530 mass spectrometric detector. 

### 2.6. Aptamer Affinity Analysis

#### 2.6.1. Flow Cytometry

The affinity of aptamers was assessed using flow cytometry on an FC-500 Flow Cytometer (Beckman Coulter, Brea, CA, USA), with data analyzed via Kaluza 1.2 software (Beckman Coulter, Brea, CA, USA). Glial tumor cell cultures were pre-treated with masking DNA (0.1 mg/mL yeast transfer RNA) in 500 µL of DPBS at 24 °C for 15 min to minimize nonspecific binding. A sample containing 150,000 cells was incubated with 100 nM FAM-labeled aptamers or ScrGli-35 as a control for 30 min at 25 °C in DPBS. FAM-labeled oligonucleotides were excited by 488 nm laser. The cells were then washed twice with 1 mL of DPBS, resuspended in 0.5 mL, and analyzed by flow cytometry within 30 min. Each experiment was conducted in triplicate, and the results are presented as the average of these measurements, with error bars indicating standard deviation.

To determine the apparent dissociation, constants ($K_{d}$s) of the aptamers’ flow cytometry was utilized. The primary culture or glial tumor cells obtained from patients were exposed to various concentrations of FAM-labeled aptamers, specifically 2 nM, 6 nM, 10 nM, 20 nM, 30 nM, 60 nM, 80 nM, 100 nM, 150 nM, and 300 nM. The resulting data were processed using Kaluza 1.2 software. The analysis involved plotting regression affinity curves, which depicted the relationship between the percentage of glial tumor cells bound to the aptamers and the corresponding aptamer concentrations.

This analysis was grounded in the principles of the Michaelis–Menten equation, under the premise of excess aptamer concentration:

$$\frac{\left[{RL}_{inf}\right]}{\left[R_{0}\right]}=\frac{\left[L_{0}\right]}{[L_{0}]+K_{d}},$$

where

$\left[R_{0}\right]$ is the concentration of the lung cancer cells;

$\left[L_{0}\right]$ is the concentration of the aptamers;

$\left[{RL}_{inf}\right]$ denotes the equilibrium concentration of the complex formed between the lung cancer cells and aptamers;

$K_{d}$ is the dissociation constant of the aptamers;

$\frac{\left[{RL}_{inf}\right]}{\left[R_{0}\right]}$ is the fraction of bound cells in a steady state.

A hyperbolic regression model was constructed based on the equation $F\left(\left[L_{0}\right]\right)=\frac{\left[L_{0}\right]}{[L_{0}]+K_{d}},$ using the set of measured data points, which represented the fraction of bound cells $\frac{\left[{RL}_{inf}\right]}{\left[R_{0}\right]}$ as a function of the aptamer concentration [$L_{0}]$. This allowed for the determination of the $K_{d}$ value from the plotted curve.

#### 2.6.2. Fluorescence Polarization Binding Dynamics

Fluorescence polarization (FP) is a fluorescence-based detection method widely used to monitor molecular interactions in solution. FP is typically used to assess biomolecular interactions such as protein–protein and protein–DNA binding, as well as enzyme activity. Low levels of polarization (P) indicate that the fluorescently labeled aptamers remain unbound and rotate rapidly in solution, emitting unpolarized light. High P levels indicate the presence of larger molecular complexes, such as the binding of aptamers to cells. The increased molecular volume slows down the aptamer’s rotation and results in the emission of predominantly polarized light. FP is calculated by the following equation:

$$P=(F_{∥}-F_{⊥})/(F_{∥}+F_{⊥})$$

where $F_{∥}$ indicates the fluorescence intensity parallel to the excitation light plane and $F_{⊥}$ is the fluorescence intensity perpendicular to the excitation light plane.

The Clariostar Plus microplate reader (BMG LABTECH, Ortenberg, Germany) was used for FP measurements. The optical system had the following settings: excitation (635 ± 10) nm, dichroic longpass filter (LP) 659 nm, emission (680 ± 10) nm, and target polarization (P) value 30 mP (mP = P/1000). The number of flashes in the kinetic window was equal to 50 for each point measurement. The measurements were performed at room temperature.

FP was measured in the plate mode. In this mode, all wells of the plate were sequentially scanned, which enabled the observation of the signal change in all wells simultaneously. Initially, 80 µL of Cy5-labeled Gli-55, Gli-35, and ScrGli-35 with a concentration of 125 nM were injected into the wells. First, the aptamer signal without cells was measured, then 20 µL of tumor cells were injected into all wells, and the mixing process was started at 200 rpm for 20 s. After this, the measurement of the FP signal continued. After adding cells to the well of the plate, the aptamer concentration became equal to 100 nM. The kinetics of the FP changes were measured for 60 min after cell injection. The FP data are presented as blank-corrected, meaning that the background (the medium with no cells or aptamers) has been subtracted from each measurement point.

### 2.7. BBB Model

The study utilized a four-cell model of the BBB in vitro [16]. This model consisted of four types of cells: neurons, astrocytes, endothelial cells, and glial tumor cells. The progenitor cells used to derive neurons and astrocytes were sourced from the brains of newborn rats (4 days postnatal). These cells were extracted from the brains of newborn rats in ice-cold phosphate-buffered saline (PBS) containing 2% glucose and were cultured in NeuroCult^®^ NS-A Proliferation Medium (StemCell, Vancouver, BC, Canada) in a humidified atmosphere with 5% CO_2_ at 37 °C. The medium was changed every 2 days. Once a sufficient number of neurospheres (100–150 µm in diameter) were obtained, they were differentiated into astrocytes in Astrocyte Medium (ScienCell, Carlsbad, CA, USA) and into neurons in Neuronal Medium (ScienCell, USA).

Endothelial cells were isolated from the blood vessels of the rat brain parenchyma (10 days postnatal) following a specific protocol [17]. All procedures were conducted on ice under sterile conditions.

The differentiated cells were phenotyped using monoclonal antibodies against GFAP (BioLegend, San Diego, CA, USA), neuron-specific enolase (NSE) (AbCam, Waltham, MA, USA), and the endothelial marker ZO1 (Santa Cruz Biotechnology Inc., Dallas, TX, USA). The cells (astrocytes, neurons, and endothelial cells) were organized into monolayers to form an in vitro BBB model in a 12-well culture plate with inserts (pore size: 0.4 µm, insert diameter: 12 mm, Corning, Corning, NY, USA). The astrocyte–neuron co-culture (300,000 cells/well) was seeded at the bottom of each well (total cell count 3 × 10^5^, culture medium volume 1.3 mL), and the brain endothelial cells were seeded into the inserts (cell count 2 × 10^5^, culture medium volume 0.7 mL). The cultures were maintained in DMEM supplemented with 10% FBS, glutamine, and antibiotics (penicillin and streptomycin). Two days after model formation, a monolayer of cells was observed at the bottom of the well and in the insert.

After the formation of monolayers in the wells and inserts, the inserts containing endothelial cells were placed into the wells containing astrocytes and neurons at the bottom. The following day, the primary cell culture of the glial tumor was added to the bottom layer. Cy5-labeled aptamers Gli-55, Gli-35, and an ScrGli-35 were added to the inserts at a concentration of 500 nM. Fluorescence levels in the inserts and wells were measured using a Clariostar Plus microplate reader (BMG LABTECH, Ortenberg, Germany) with excitation at 635 ± 10 nm and emission at 680 ± 10 nm before the addition of aptamers immediately after their addition and 24 h post addition. Fluorescence microscopy (excitation at 488 nm) was used to visualize the binding of FAM-labeled Gli-35 to glial tumor oncospheres.

### 2.8. Confocal Laser Scanning Microscopy (CLSM)

Cell cultures in glass-bottom wells were incubated with Cy5-labeled aptamers Gli-55 and Gli-35 at a final concentration of 100 nM for 30, 60, 120, and 240 min. Additionally, the cells were stained with nucleus staining dye DAPI (4′, 6-diamidino-2-phenylindole) and membrane potential DiBAC_4_(3) (Bis-(1,3-Dibutylbarbituric Acid) Trimethine Oxonol). Cy5 labels were attached to the aptamers at the 5′ prime region during synthesis by click chemistry. CLSM binding analyses were conducted using an LSM 780 NLO Confocal microscope (Carl Zeiss, Hamburg, Germany), with image processing performed using ZEN2 v. 2011 (Carl Zeiss, Hamburg, Germany) software. Cy5-labeled aptamers were excited by the 640 nm laser.

## 3. Results and Discussion

### 3.1. Aptamer Structure Optimization

**Figure 1:**
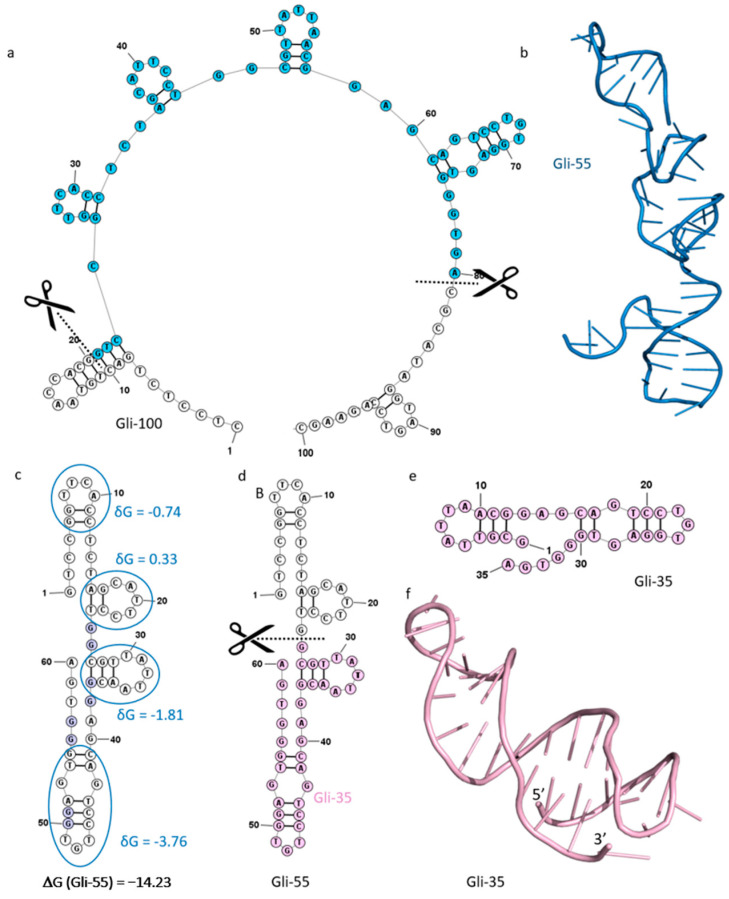
Truncation of the full-length aptamer. (**a**) The secondary structure of the Gli-100 aptamer and the scheme of the truncation, the blue color shows a Gli-55 aptamer sequence; (**b**) the tertiary structure of Gli-55; (**c**) the secondary structure of the Gli-55 aptamer with the folding energies of its structural elements; (**d**) the scheme of the Gli-55 truncation, the pink color shows a new Gli-35 aptamer; (**e**) the secondary structure of Gli-35; (**f**) the tertiary structure of Gli-35 obtained after MD simulations.

The previously described aptamer Gli-55 effectively binds to GBM [7]. This aptamer was obtained by truncating the full-length 100 nucleotide aptamer with the sequence: 5′-CTC CTC TGA CTG TAA CCA CGG TCC GGT TCA CCT CTA GCA TTC CTG GCG TTA TTA ACG GAG CAG TCC TGT GGA GTG GGT GACG CAT AGG TAG TCC AGA AGC-3′ (Figure 1a,b). Removing the nucleotides from the 5′ and 3′ prime regions did not influence aptamer affinity to glial tumor cells. The aptamer consists of 60 nt with the sequence: 5′-GTCCG GTTCA CCTCT AGCAT TCCTG GCGTT ATTAA CGGAG CAGTC CTGTG GAGTG GGTGA-3′. It was used for the diagnosis and visualization of brain tumors [7]. Nevertheless, Gli-55 has a nonspecific binding of around 7%, which might be crucial for therapeutic applications [7]. The 60 nt aptamer is still too long for use as a drug. Shortening it will reduce the cost of synthesis and facilitate its internalization into glial cells. Thus, we made an attempt to truncate it.

**Figure 2:**
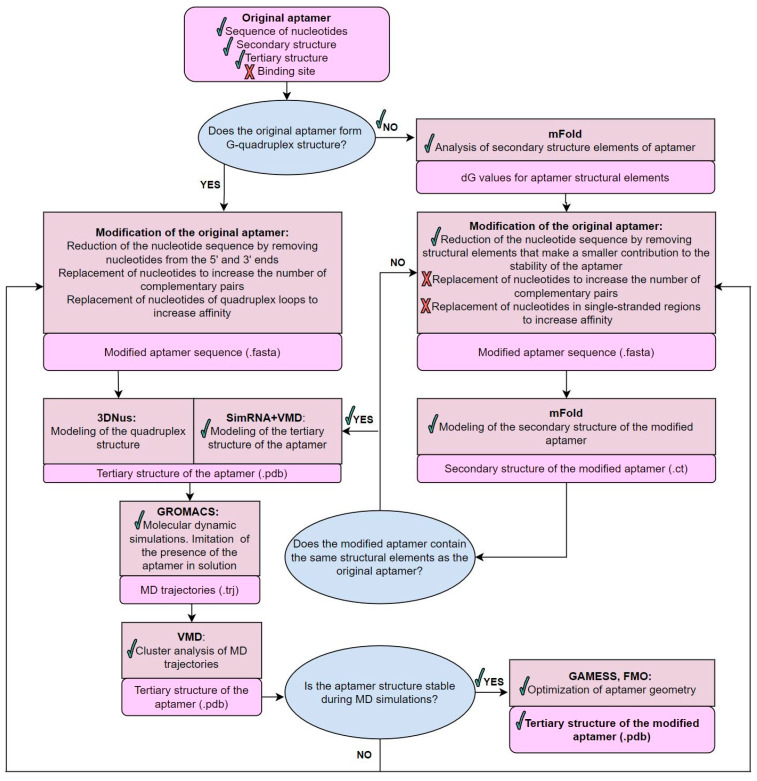
General scheme of aptamer modification, including information on the construction of three-dimensional models and the evaluation of their stability.

The truncation of the Gli-55 aptamer was performed according to the scheme presented in Figure 2.

The truncation of Gli-55 was based only on its structure peculiarities. At first, we analyzed if the original aptamer forms a G-quadruplex structure. Despite the fact that Gli-55 contained four couples of guanines (Figure 1c), circular dichroism showed that the aptamer did not form a G-quadruplex [7]. Therefore, the secondary structure of the Gli-55 aptamer was analyzed using the mFold web server. This program provides the free energy of aptamer folding and allows one to estimate the contribution to the free energy for each structure fragment of the aptamer. Figure 1c shows the free energy of Gli-55 folding and energy contributions for different hairpins of the aptamer. Analysis showed that the harpins formed by 27–37 and 41–55 nucleotides contributed more than the two other hairpins. Thus, nucleotides from 1 to 25 were removed from the sequence (Figure 1d). Since the original Gli-55 aptamer did not form a G-quadruplex, we prevented this possibility for a new truncated aptamer by reducing the sequence by one nucleotide, guanine, from the 5′-end.

Then, the mFold web server was used to confirm that the secondary structure of the truncated aptamer formed the same structure elements as the corresponding part of the full-length initial aptamer (Figure 1e). Therefore, a sequence of 35 nt was obtained, and the new shortened aptamer was called Gli-35 (Figure 1e,f).

At the next stage, the corresponding tertiary structure of the aptamer was designed with a combination of the SimRNA and VMD programs. In the case of a G-quadruplex structure, the 3DNus web server [18] would be used for the modeling of the G-quartet with corresponding loops. The obtained 3D model was subjected to 200 ns MD simulations followed by the clustering analysis of the MD trajectory. The obtained atomic structure of the Gli-35 aptamer is shown in Figure 1f.

Since the designed truncated aptamer had the same structural elements as the initial aptamer and its hairpins were stable during MD simulations, which imitate the presence of the aptamer in the solution, the proposed Gli-35 aptamer was subjected to further investigations in vitro.

### 3.2. Analyses of Aptamer Properties

**Figure 3:**
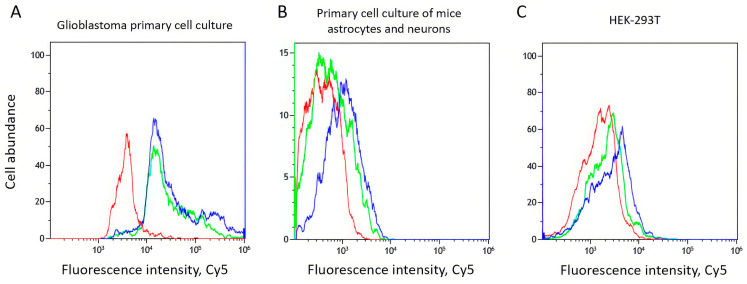
The flow cytometry binding analysis of Gli-55 (blue curve) and Gli-35 (green curve) in comparison with the intact cells (red curve) to primary IDH1 mutant glioma cell culture (**A**), the primary cell culture of astrocytes and neurons (**B**), and the HEK-293T cell line (**C**).

To evaluate the effectiveness of the aptamer structure optimization, both the affinity and specificity of the initial and truncated aptamers were assessed. All measurements were performed in triplicate. The binding of the initial Gli-55 aptamer to primary IDH1 mutant GBM cells was slightly higher than that of the shortened version. Thus, the deletion of several nucleotides only slightly affected its binding to the target cells (Figure 3A). Meanwhile, the selectivity of the Gli-35 aptamer, assessed using a primary astrocyte cell culture (Figure 3B) and the HEK cell line (Figure 3C), increased. This enhanced selectivity is very important for the development of targeted therapies.

**Figure 4:**
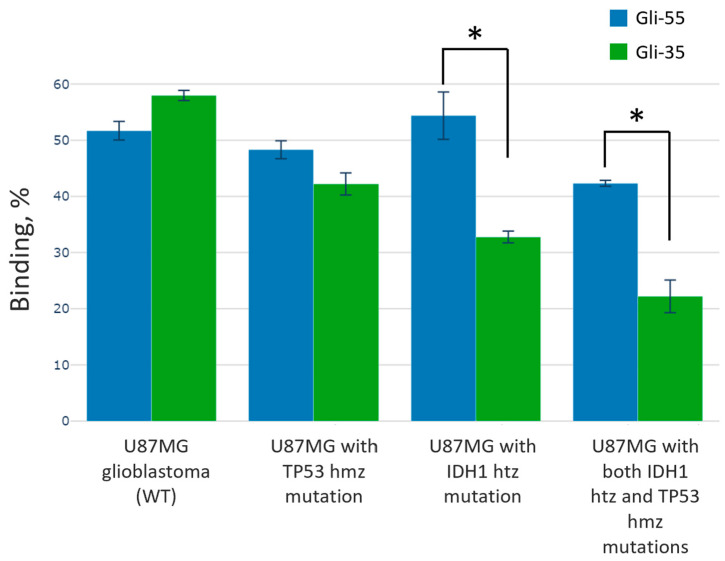
Binding level of Gli-55 (blue bars) and Gli-35 (green bars) to cell cultures: wild type U87MG GBM, U87MG GBM with TP53 hmz mutation, U87MG GBM with IDH1 htz mutation, and U87MG GBM with both IDH1 htz and TP53 hmz mutations. The asterisk (*) indicates statistically significant differences (*p*-value < 0.05) in aptamer binding.

To assess whether the aptamers are sensitive to various genotypes typical for glial tumors, we tested their binding to four glioma cell lines at a low aptamer concentration (50 nM) to better observe differences between the WT, TP53 hmz mutant, IDH1 htz mutant, and a combination of both mutations: IDH1 htz and TP53 hmz. The truncated and long aptamers exhibited nearly the same binding rate with WT GBM and U87 TP53 hmz mutant GBM (Figure 4). In this experiment, Gli-35 demonstrated a lower affinity for glial tumor cells with the IDH1 htz mutation and showed slightly less sensitivity to U87MG GBM with the IDH1 htz mutation and U87MG GBM with both the IDH1 htz and TP53 hmz mutations compared to its longer predecessor, Gli-55. Overall, the binding rates of these aptamers are similar. The apparent dissociation constant measured for U87MG GBM with the IDH1 htz mutation (*K*_*d*_) of Gli-55 is 12.2 nM, while for Gli-35, it is 13.6 nM. These values are similar to the *K*_*d*_s of other reported aptamers [19].

### 3.3. Fluorescence Polarization

**Figure 5:**
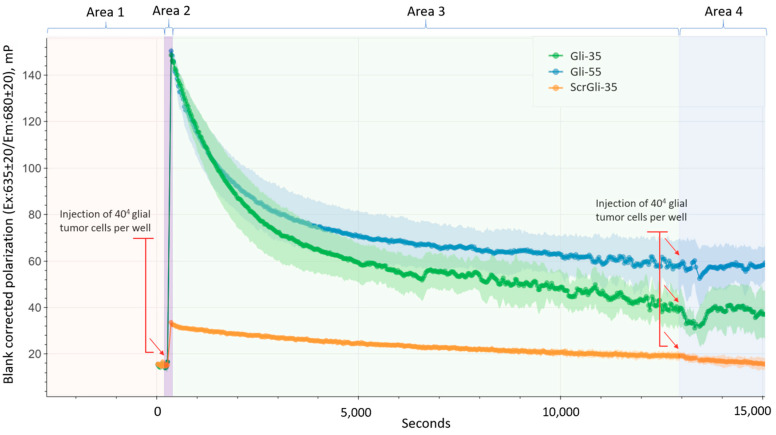
The binding dynamics curves of the aptamers to glial tumor cells obtained through FP measurements. The green curve represents the Cy-5-labeled aptamer Gli-35, the blue curve represents Gli-55, and the orange curve corresponds to a control ScrGli-35. The red labels indicate the points at which the cells were injected into the wells. A distinct average line is derived from five replicates, and variability is shown in lighter shades. Area 1 corresponds to the FP of the fluorescently labeled oligonucleotides before adding the cells. Area 2 corresponds to aptamer binding dynamics to glial tumor cell membranes. The changes in Area 3 indicate that the dye gained more vibrational freedom, suggesting aptamer internalization into the cells. Finally, Area 4 represents the state after the second addition of the cells.

To evaluate the binding dynamics of the Gli-55 and Gli-35 aptamers, FP analysis was performed. Figure 5 displays the binding dynamics curves for the aptamers with target cells, featuring an average line derived from five replicates, with variability indicated by lighter shades.

For the measurements, we use an excess of aptamers relative to the cells. Consequently, the anisotropy (degree of polarization) of the aptamers is initially unstable and may decrease due to aggregation. Therefore, after adding the aptamers to the buffer in the plate well, they were allowed to stabilize before measurements began, once the polarization had reached a plateau. The initial FP (anisotropy) is relatively low, at about 20 mP (Figure 5 area 1).

Upon the introduction of glial tumor cells with the IDH1 htz mutation (indicated by the red arrow) into the wells containing the fluorescent Cy-5 labeled aptamers (Gli-55 is shown in blue, Gli-35 in green, and a control nonspecific oligonucleotide in orange), there was a sharp increase in FP. This suggests that the specific binding of the aptamers Gli-55 and Gli-35 with the Cy-5 label occurs (Figure 5, area 2, represented by the blue and green curves), which reduces the vibrational and rotational freedom of the label molecule, leading to the depolarization of the emission. Consequently, the degree of polarization significantly increases, indicating that the oligonucleotides bind to the cells (Figure 5). We hypothesize that, at this stage, the aptamers bind to cell membrane proteins, as we previously observed with the longer Gli-100 and Gli-55 aptamers [20]. A slight increase in anisotropy for ScrGli35 (Figure 5 area 2, orange curve) may be due to partial the nonspecific binding or sorption of the fluorescent label on the surface of the oligonucleotide. Additionally, an increase in the solution’s viscosity may also contribute to this effect.

The subsequent decrease in FP for aptamers Gli-55 and Gli-35 (Figure 5, area 3, blue and green curves) indicates that the dye gained more vibrational freedom and suggests either the detachment of the aptamers from the cells, the desegregation of glial tumor cells due to aptamer binding, or the internalization of the aptamers into the cells. To investigate this, we added an additional portion of glial tumor cells to determine if any unbound aptamers remained in the solution. The second addition of glioma cells caused a disturbance and instability in polarization due to the injection of cells, which then stabilized and did not significantly alter the FP. This indicates that all free aptamers in the PBS solution have entered the cells.

Therefore, we assume that the decrease in FP was caused by the internalization of the aptamers into the cells. As more aptamers enter the cells, the FP value decreases. This reduction in polarization indicates that the aptamers with the dye gained more vibrational freedom inside the cells. However, it is also possible that this is related to optical effects from the cell membranes or physiological processes within the cytoplasm. Based on the rate of decline in the graphs, aptamer Gli-35 enters the cells more rapidly than aptamer Gli-55, probably because of its size.

The FP of the control ScrGli-35 showed only a slight increase, indicating a lack of specificity for these cells, followed by an insignificant decrease, thus reflecting a low level of internalization.

### 3.4. Confocal Laser Scanning Microscopy

**Figure 6:**
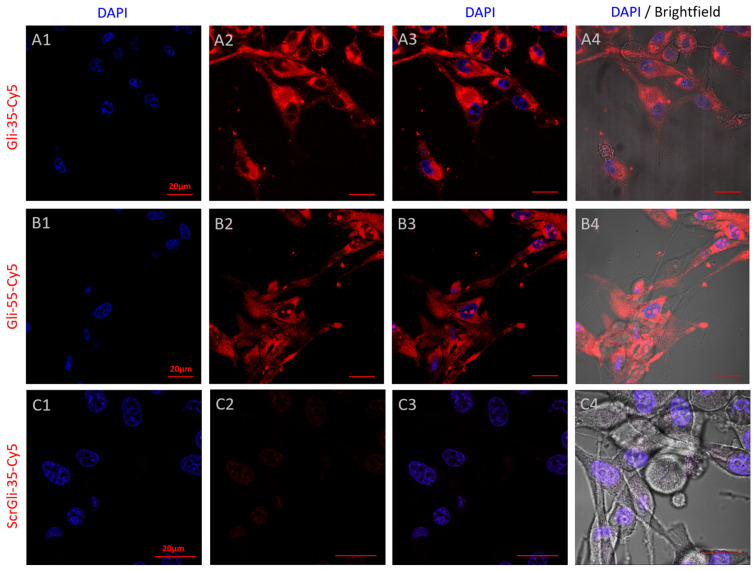
A comparison of Gli-35-Cy5 (**A**) and Gli-55-Cy5 (**B**) aptamer binding to IDH1 htz mutant GBM cells in culture, shown in confocal laser scanning microscopy. A scrambled control aptamer ScrGli-35-Cy5 (**C**) shows minimal binding. The images include DAPI nuclear staining (**A1**,**B1**,**C1**), Cy5 staining (**A2**,**B2**,**C2**), DAPI and Cy5 staining overlays (**A3**,**B3**,**C3**), DAPI, Cy5 and brightfield overlays (**A4**,**B4**,**C4**). Magnification 100×. Scale bars 20 µm.

To confirm the findings obtained from the FP experiments, prove aptamer internalization, and obtain visual confirmation, we performed confocal laser scanning microscopy. The Cy5-labeled Gli-35 aptamer was incubated with glial tumor cells in culture for one hour. After the incubation period, the cells were visualized using a confocal laser scanning microscope (Figure 6). The images showed that both the Gli-55 and Gli-35 aptamers were absorbed by the primary culture of glial tumor cells with the IDH1 heterozygous mutation and accumulated in the cytoplasm, but not in the nucleus. In contrast, the scrambled ScrGli-35 did not show any significant specific binding, but it was absorbed into the nucleus.

**Figure 7:**
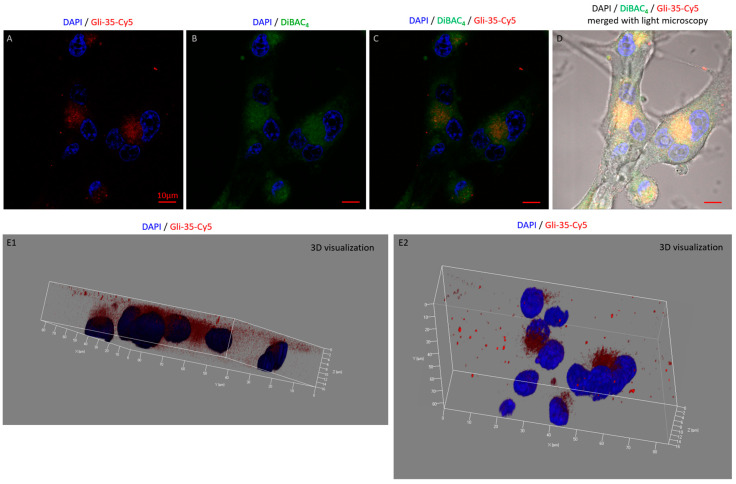
Gli-35 internalizes inside glial tumor cells in culture (IDH1 htz mutant GBM). The confocal laser scanning microscopy of cells stained with nucleus staining dye DAPI and Cy5-labeled aptamer Gli-35 (**A**); DAPI and membrane potential DiBAC_4_(3) (for membrane indicating) dyes (**B**); DAPI, DiBAC_4_(3), and Gli-35-Cy5 (**C**); DAPI, DiBAC_4_(3), and Gli-35-Cy5 merged with light microscopy T-PMT (a transmitted detection module with a photon counting PMT) (**D**); 3D representation of the cells with DAPI-stained nuclei and Cy5-labeled aptamer Gli-35 (**E1**,**E2**). Magnification 100×. Scale bars 10 µm.

Indeed, the confocal laser scanning microscopy (CLSM) effectively demonstrated the internalization of aptamers into the cells. Following a 30 min incubation period, the aptamers, labeled with Cy5, concentrated near the nucleus, as shown in Figure 7A and the 3D visualizations in E1 and E2. This proximity suggests that the aptamers may be involved in transport mechanisms near the nucleus. Additionally, the images in Figure 7B–D reveal that the aptamers also localize under the cell membrane stained with the DiBAC_4_ (green fluorescence) near the nuclear DAPI stain (blue). The 3D visualizations in Figure 7(E1,E2) add depth, illustrating the spatial arrangement and concentration gradient of the aptamers within cellular compartments, which could be crucial in terms of contributing to mechanistic insights into how aptamers accumulate in glial tumor cells. Figure 7C shows the merged presence of the Cy5-labeled aptamer (red) with the DiBAC_4_(3) staining (green), creating a composite visualization of the cellular internal environment. Thus, the CLSM results supported the conclusion drawn from the FP findings regarding the internalization of aptamers into the cells.

**Figure 8:**
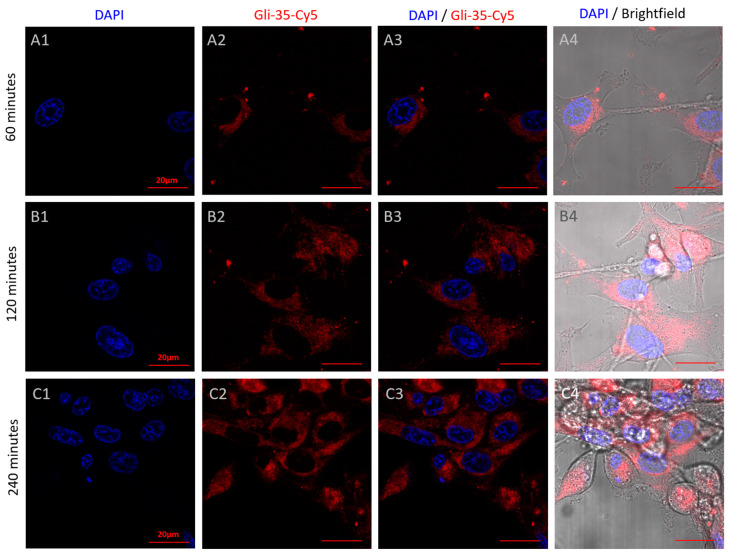
Binding dynamics of the Gli-35-Cy5 aptamer, shown in confocal laser scanning microscopy. Images were taken after 60 (**A**), 120 (**B**), and 240 (**C**) minutes of incubation with Gli-35-Cy5 (**A2**,**B2**,**C2**), DAPI nuclear staining (**A1**,**B1**,**C1**), overlay of Gli-35 and DAPI (**A3**,**B3**,**C3**), overlay of Gli-35 and DAPI with brightfield (A**4**,**B4**,**C4**). Magnification 100×. Scale bars 20 µm.

FP demonstrated the rapid binding of aptamers to the cell membrane, immediately initiating internalization (Figure 5). This process resulted in a reduction in FP due to the vibrational freedom of the aptamer–dye complex, and indeed after 30 min of incubation, the dye was observed to be uniformly distributed around the nucleus and not localized to any specific area (Figure 7). To further understand the dynamics of aptamer accumulation and its ability to bind to intracellular targets, we extended the incubation of Gli-35 to 60, 120, and 240 min (Figure 8). Interestingly, after one and two hours of incubation, the aptamers accumulated inside cells within small vesicles of 0.5–1 µm in diameter (Figure 8A,B and Figure S1). However, after four hours, the aptamers bound to certain structural elements (Figure 8C). Previously, using the AptaBiD technique, we identified and validated—using antibodies—a post-translationally modified form of GFAP as one of the most likely targets of the full aptamer Gli-55 [7]. GFAP, an intermediate filament protein expressed by astrocytes, is a promising target for specific anti-glial tumor therapy.

Overall, these observations underscore the potential role of Gli-35 aptamers in targeting specific cellular compartments, which could enhance therapeutic strategies aimed at glial tumor cells.

### 3.5. Assessment of the Aptamer’s Ability to Penetrate the BBB

**Figure 9:**
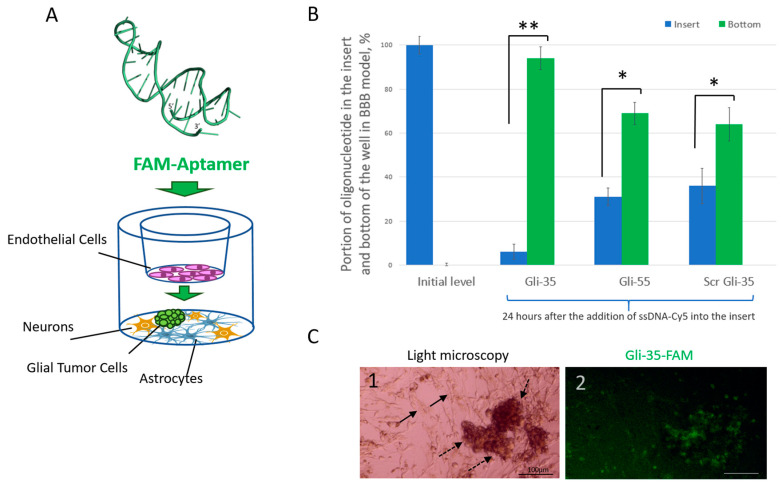
Migration of fluorescently labeled aptamers through the in vitro blood–brain barrier model. A schematic representation of the in vitro four-cell blood–brain barrier model is used for the assessment of aptamer transfer. (**A**) The model comprises four types of cells: a monolayer of endothelial cells in the inserts with 0.4 µm pores in a Transwell plate, neurons, astrocytes, and glial tumor cells at the bottom. (**B**) Microplate reader data for the aptamers and scrambled oligonucleotide indicate the relative level based on the combined fluorescence signal from both the well bottom and the insert. Statistically significant differences in aptamer presence between the insert and the bottom of the well are indicated by asterisks: * *p* < 0.05; ** *p* < 0.01. (**C**) Light (**C1**) and fluorescence microscopy (**C2**) images of glial tumor cells incubated with the FAM-labeled Gli-35 aptamer. The oncospheres are indicated by dashed arrows and are cocultured on a layer of neurons and astrocytes, which are marked by black arrows. Magnification 20×. Scale bars 100 µm.

The ability of both the initial and truncated aptamers to cross the BBB was evaluated using a four-cell in vitro BBB model. Cy5-labeled aptamers Gli-55 and Gli-35, along with a scrambled Gli-35 oligonucleotide used as a control, were introduced into the inserts (with a pore size of 0.4 µm) containing a monolayer of endothelial cells in a 12-well culture plate. This setup featured an astrocyte–neuron co-culture at the bottom of the wells (Figure 9A).

As a control to verify the functionality of the model, methylene blue and trypan blue were added to the plate. Normally, trypan blue does not pass through the blood–brain barrier [21], while methylene blue does [22]. Experiments with these two dyes indicated that the model is valid and can be used for the assessment of the ability of the aptamers to cross the BBB (Figure S2). Indeed, in this model, trypan blue remained in the insert (Figure S1A), while methylene blue migrated to the bottom of the well and stained the cells (Figure S1B).

The aptamers added into the inserts and were allowed to diffuse into the bottom layer (Figure 9A) over a period of 24 h post addition. The results indicated that Gli-55, Gli-35, and scrambled Gli-35 were able to penetrate the BBB in the four-cell BBB model; however, Gli-35 achieved the highest success, as shown in Figure 9B. These results are consistent with the data we obtained previously in mice, showing that aptamers are capable of crossing the BBB into the mouse brain [20]. Furthermore, in a four-cell model, where glial tumor cells were co-cultured with neurons and astrocytes in the bottom layer (Figure 9(C1)), Gli-35 selectively stained the glial tumor oncospheres (Figure 9(C2)).

## 4. Conclusions

The pharmaceutical industry is showing growing fascination with therapeutic oligonucleotides. Currently, 21 oligonucleotide-based medications have received commercial approval in both the US and EU, comprising 2 aptamers [23], 12 antisense oligonucleotides (ASOs), 6 small interfering RNAs (siRNAs), and a combination of single-stranded and double-stranded polydeoxyribonucleotides [24]. In addition, hundreds more are at various stages of clinical and preclinical development [25]. However, the current methods for synthesizing long oligonucleotides, particularly aptamers, pose significant production challenges.

The study highlighted the efficacy of employing molecular modeling supported by experimental procedures for aptamer structure elucidation to alter the nucleotide sequence of an aptamer while maintaining its binding capabilities. By excising nonfunctional regions from the oligonucleotide sequence, nonspecific binding is minimized, and binding dynamics and internalization are enhanced. By using modeling, we can lower the costs of producing the therapeutic agent and also avoid the expense of finding the best nucleotide sequence through a trial-and-error process. This strategic approach not only boosts efficiency but also conserves resources, making it an advantageous method for aptamer development. In this work, truncation of Gli-55 aptamer, which selectively binds to GBM, was performed based on the structure peculiarities of the original aptamer.

The molecular design showed that the truncated aptamer formed the same structure elements as the original sequence. The new truncated aptamer was called Gli-35 and its binding affinity was studied in vitro. Since Gli-35 demonstrated a binding affinity comparable with the affinity of the original Gli-55, the described approach to aptamer truncation can be used for similar cases where information about the molecular target is not available.

## Data Availability

Upon request.

## Supplementary Materials

The following supporting information can be downloaded at: https://www.mdpi.com/article/10.3390/cancers16234111/s1, Figure S1: The aptamer Gli-35-Cy5 accumulated in the small vesicles 0.2-1µm, shown in confocal laser scanning microscopy. Images were taken after 60 min of incubation with Gli-35-Cy5 (1), overlay Gli-35 and DAPI nuclear staining with brightfield (2). Magnification 100×. Scale bars 10 µm; Figure S2: Verification of the functionality of the in vitro four-cell blood-brain barrier model used for the assessment of aptamer transfer. Normally, trypan blue does not pass through the blood-brain barrier (A), while methylene blue does (B). The schematic representation is shown in panel 1. Light microscopy images of the inserts with endothelial cells and added dyes are presented in panel 2. Panel 3 shows the bottom of the wells with a coculture of neurons, astrocytes, and glial tumor cells.
