# Supplementary material for: Aptamer’s Structure Optimization for Better Diagnosis and Treatment of Glial Tumors
Source: Cancers (Basel). 2024 Dec 8;16(23):4111. doi: 10.3390/cancers16234111 (PMC11640682; doi:10.3390/cancers16234111)
Supplement: Supplementary file 1 [file cancers-16-04111-s001.zip › cancers-3331572-supplementary.pdf]

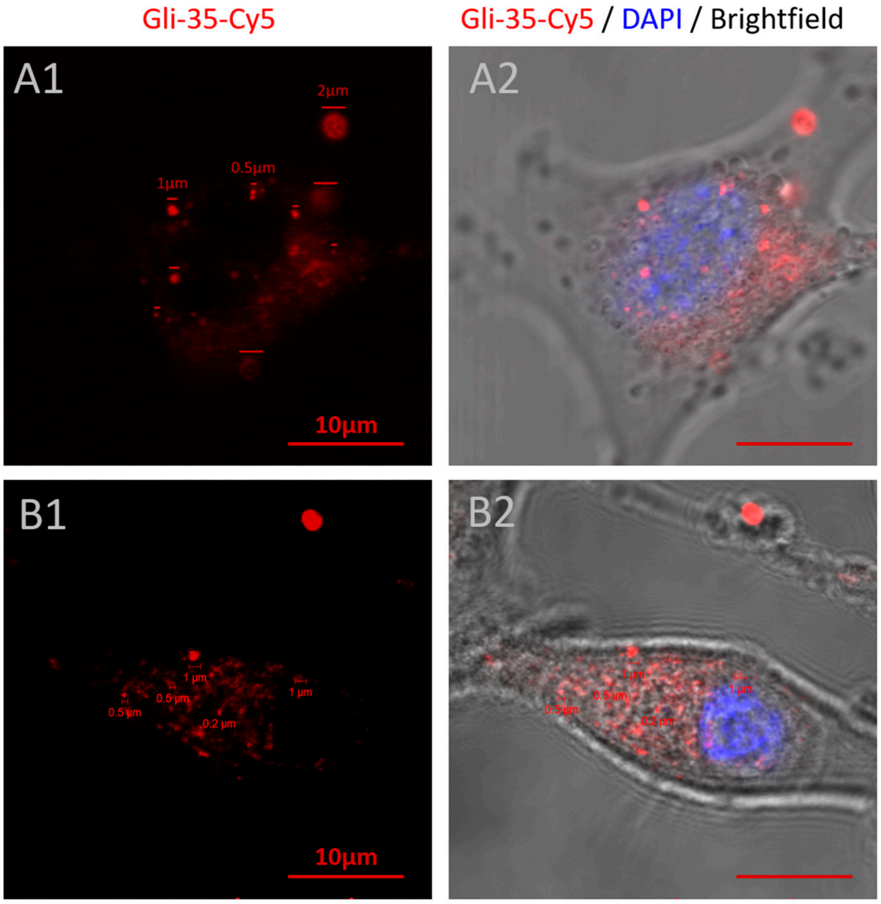

Figure S1. The aptamer Gli-35-Cy5 accumulated in the small vesicles 0.2-1μm, shown in confocal laser scanning microscopy. Images were taken after 60 minutes of incubation with Gli-35-Cy5 (1), overlay Gli-35 and DAPI nuclear staining with brightfield (2). Magnification 100×. Scale bars 10 μm.

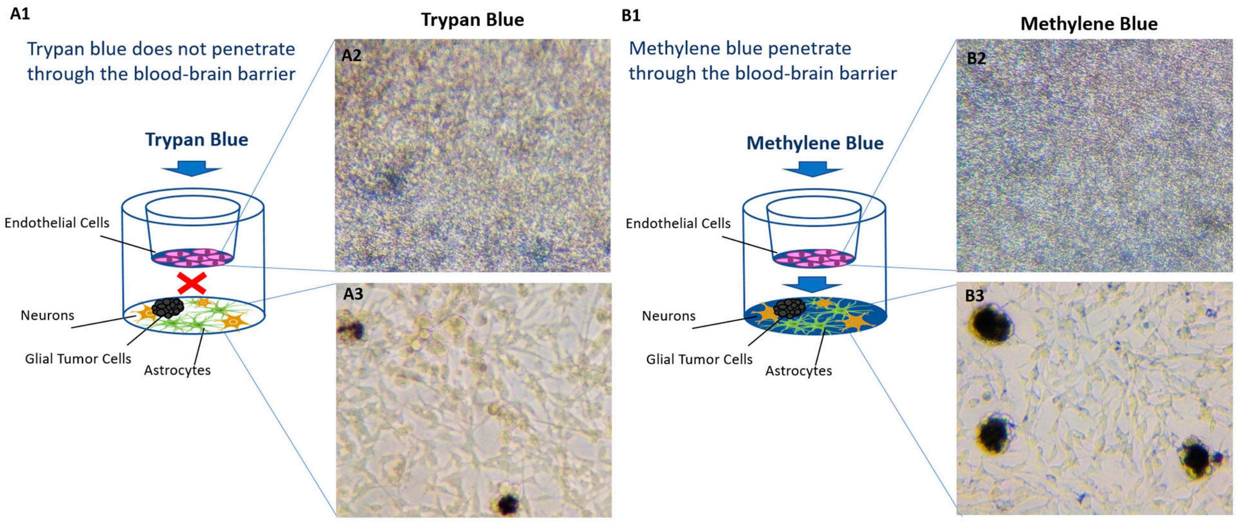

Figure S2. Verification of the functionality of the *in vitro* four-cell BBB model used for the assessment of aptamer transfer. Normally, trypan blue does not pass through the blood-brain barrier (A), while methylene blue does (B). The schematic representation is shown in panel 1. Light microscopy images of the inserts with endothelial cells and added dyes are presented in panel 2. Panel 3 shows the bottom of the wells with a coculture of neurons, astrocytes, and glial tumor cells.
